# Navigating cancer treatment in people with intellectual disability: a qualitative study of professionals’ and family members’ perspectives

**DOI:** 10.1186/s12885-026-15601-6

**Published:** 2026-01-30

**Authors:** Daniel Satgé, Kristopher Lamore, Sarah Habib-Hadef, Aurélie Curnier, Hélène Carbone, Florence Cousson-Gélie

**Affiliations:** 1 Oncodéfi, Parc Euromédecine, 209 Avenue des Apothicaires, Montpellier, 34090 France; 2 INSERM UA11 Institute Desbrest of Epidemiology and Public Health, INSERM, Univ Montpellier, 641 Avenue du Doyen Gaston Giraud, Montpellier, 34093 France; 3 Univ. Lille, CNRS, UMR 9193 - SCALab - Sciences Cognitives et Sciences Affectives, Lille, F-59000 France; 4 Univ Montpellier Paul Valéry, EPSYLON EA 4556, Montpellier, F34000 France; 5 Epidaure Prevention Department, Montpellier Cancer Institute, 208 Avenue des Apothicaires, Parc Euromédecine, Montpellier, F34000 France

**Keywords:** Cancer treatment, Diagnosis delay, Handicap, Intellectual disability, Medical education, Obstacles, Qualitative research

## Abstract

**Background:**

Cancer occurs in people with intellectual disability (ID) as frequently as in the general population. However, successful cancer treatment is less likely in people with ID, and the underlying reasons are not well-documented. Here we investigated the perspectives of health care professionals (HCPs) and family members regarding cancer care delivery and challenges for people with ID, with the aim of identifying the barriers and facilitators to care.

**Methods:**

Semi-structured interviews were conducted with professionals and family members involved in cancer care for adults with ID. The obtained data were subjected to thematic analysis.

**Results:**

Thirty-seven interviews were analyzed. Ten barriers to care were identified, four of which emerged from the participants’ accounts. The main identified barriers to care were related to patients’ limitations (psychological, physical, and treatment non-agreement), communication difficulties (i.e., with the patient, or between professionals or institutions), professionals’ lack of knowledge, and advanced cancer stage at diagnosis. Facilitating circumstances were also identified—which included any presence of a proactive family member or professional caregiver, presence of a proactive family member or caregiver throughout the course of treatment, the patient having a confident and positive attitude, and tailored environment.

**Conclusion:**

While barriers related to patient characteristics are unmodifiable, it may be possible to improve professionals’ knowledge, communication skills, the patient’s environment, and diagnostic delays. Thus, many obstacles that complicate cancer treatment for people with ID, and those who care for them, can be alleviated to potentially improve cancer prognosis in this vulnerable population.

**Supplementary Information:**

The online version contains supplementary material available at 10.1186/s12885-026-15601-6.

## Introduction

Intellectual disability (ID) is a neurodevelopmental condition characterized by significant cognitive impairments that affect daily functioning [1, 2]. People with ID experience various challenges—including difficulties in learning, abstract reasoning, problem solving, and adaptative behaviors [1]. ID is typically identified during childhood, and can stem from genetic factors (e.g., Down syndrome, Fragile X syndrome, and other chromosomal abnormalities) or be acquired, for example, due to fetal alcohol syndrome, severe head trauma, or birth-related oxygen deprivation [3]. The diagnostic criteria for ID include intelligence quotient scores (< 70) and adaptive functioning assessments, which are also used to determine the level of severity [1]. Current epidemiological data indicate that ID affects 1–3% of individuals in developed countries, while up to 13% of individuals have similar support needs but do not meet the full diagnostic criteria for ID [4, 5]. Thus, there is a great need for comprehensive support systems, including early interventions and specialized education, to enhance outcomes. These support needs are particularly critical when people with ID face additional health challenges, such as cancer diagnosis and treatment.

Adults with ID develop cancer at rates comparable to those of the general population [6–9], but face significant disparities in accessing cancer-related care, and more commonly exhibit poor outcomes [10, 11]. For instance, during treatment for testicular cancer, the risk of mortality is four-fold higher among men with ID, compared to men in the general population [12]. This may be related to the lack of rigorous scientific evidence regarding effective cancer treatment for people with ID. To improve care for these patients, there is a need for more detailed information about cancer management in this population [10, 11, 13]. While people with ID likely require specialized attention during cancer treatment [14], they reportedly have lower hospitalization rates [15] and are less likely to receive aggressive cancer therapies [13], compared to the general population with cancer. These discrepancies suggest that patients with ID may not receive adequate or appropriate treatment.

Indeed, people with ID and cancer face significant barriers to effective diagnosis and treatment—contributing to delayed diagnoses, underutilization of preventive and palliative services, and poorer health outcomes, compared to the general population [16–19]. Delayed diagnoses in these patients can result in diagnosis of cancer at an advanced stage, which limits treatment options and leads to worse outcomes [20–22] People with ID also commonly face additional obstacles with regards to having their specific needs and preferences recognized, and incorporated into care decisions [23]. Communication difficulties may impact symptom assessment, which can lead to inadequate pain and symptom management [19, 24]. Additional barriers to accessing cancer care may be that clinicians have limited training in managing cancer among people with ID, a lack of collaboration between healthcare and social services, and insufficient expertise regarding cancer treatment in residential care facilities for adults with ID [17, 18, 25]. Moreover, the quality of care can be further reduced if healthcare professionals (HCPs) and professional caregivers have negative attitudes and limited understanding regarding cancer in people with ID [16–18].

Despite these challenges, there is a notable scarcity of large-scale studies that have systematically examined the frequency of these barriers and their impact on cancer treatment outcomes for people with ID. The currently available research is limited to case studies and small-sample investigations [17, 18, 26–29], possibly due to the substantial challenges of accessing this population and successfully including them in research initiatives. To overcome these research limitations, HCPs, professional caregivers, and family members can serve as valuable informants and key stakeholders. In particular, family members play a fundamental role in supporting people with ID throughout their cancer journey [30]. Essential support is provided by family members, professional caregivers, and HCPs who are involved at every stage of the care process—from the initial identification of symptoms to treatment management and follow-up care [30, 31]. Notably, professional caregivers who work in institutions offer organized settings within group homes or residential facilities, whereas home caregivers perform a variety of tasks aimed at fostering inclusive and welcoming environments [32]. Additionally, qualitative studies could be a valuable tool for exploring the care challenges faced by people with ID, and for developing clinical recommendations. Few qualitative studies have been conducted in this population, and they have primarily focused on obstacles to care, rather than on facilitating factors.

In the present study, we investigated the perspectives of HCPs, professional carers, and family members regarding cancer care delivery and challenges for people with ID, with the aim of identifying the barriers and facilitators to care.

## Materials and methods

### Study design

A qualitative study was performed, using semi-structured interviews. As emphasized by Miles and Huberman [33], qualitative methods are particularly well-suited for exploring complex phenomena. Here they were used to ensure flexibility, to prioritize the participants’ voices, and to allow the emergence of new dimensions that more accurately reflect the realities of the field—for example, through the adaptable use of the interview guide. The study methodology and results are reported following the guidelines of O’Brien et al. [34] and the Consolidated Criteria for Reporting Qualitative Research [35].

### Population

Research was conducted between 2017–2018, with the help of Oncodéfi, a French association dedicated to improving cancer diagnosis and care for people with ID, and the Epsylon laboratory of psychology (Univ. Montpellier Paul Valéry). The Oncodéfi network is mainly based in the region Occitanie (southwest of France) but includes patients from every region in France.

Individuals were eligible if: (1) they had provided care for an adult over the age of 20 with both cancer and ID, (2) were a professional (i.e., physicians, nurses, institution directors, caregivers) or a family caregiver, (3) were at least 18 years old, and (4) gave oral consent to participate. Individuals were not invited to participate if they cared for, or were family members of, only people with cancer and ID as part of a neurodegenerative disease, or a psychiatric condition without ID. Furthermore, participants were excluded from the study post-interview if they were found to have had insufficient involvement during the care process, if they could not provide sufficient details about the patient, or if their interviews provided limited information relevant to the study aim.

People with ID were not directly interviewed, to allow for inclusion of people diagnosed with a wide range of ID levels—including people with severe ID who cannot speak and who are thus challenging to include in research projects.

### Procedure

Eligible participants were identified through Oncodéfi records. For each new patient referred to Oncodéfi, the HCPs and family caregivers involved in the case management were invited to participate in this study, along with the patients’ parents. These individuals were contacted by phone (by SHH or DS) to invite them to participate in the study and were also sent an information letter. Research interviews were planned with interested individuals, which were conducted remotely or face-to-face (by SHH, AC, HC, or DS), either at the hospital, institution, or retirement home, or at Oncodéfi. To minimize potential bias due to the interview format (phone or in-person), interviewers were trained to maintain a neutral and supportive stance in both formats, and an interview guide was used (see below). The interviewers had no prior relationship with the participants before the interviews were conducted, and participants were not previously known to the interviewers. No professional or personal relationship existed prior to recruitment.

Before the interview, participants gave their oral consent to participate and for the interview to be recorded. Oral consent was audio recorded before beginning the semi-structured interview. After completing, each interview was transcribed verbatim. No prior relationship existed between the eligible participant and the researcher conducting the interview.

Semi-structured interviews were conducted following two interview guides—one for HCPs working in oncology teams, and one for other participants. These interview guides are presented in Table 1. The explored themes were as follows: announcement of the disease; the care pathway; the symptoms experienced during treatment; the involvement of family members, HCPs, or professional caregivers in care; and the knowledge about ID (for HCPs working in oncology) or opinions about how to improve cancer care for people with ID (for other participants). Information were also collected about the participants’ demographics (i.e., sex, profession, and family relationship with the patient) and patients’ diagnosis. Notably, the interview guide development was informed by difficulties commonly reported in the scientific literature [18, 36–39], and by the professional experience of the research team. Before data collection, the guide was reviewed by three professionals in the field, and their feedback was used to adjust the phrasing of certain questions, and to ensure that the content was clear, relevant, and usable for the study objectives.

**Table 1:** Interview guides (translated from French)

Interview Guide – Professionals
1. How did the cancer diagnosis unfold?
a. What was the caregiver’s role, if present?
b. What would you suggest to improve how diagnoses are communicated?
2. How was the patient’s care managed?
a. Did the care strategy need to be adjusted?
b. How did you ensure the patient understood their illness?
c. Did the patient agree to the proposed care pathway?
d. When and why was the family and professional caregiver’s involvement necessary?
3. How did you assess the patient’s symptoms?
a. How was the patient’s pain adequately addressed?
4. How did you take the patient’s preferences into account?
a. Did the patient refuse certain procedures? If so, how was this issue resolved?
b. What was the greatest difficulty you encountered?
5. What adjustments would you suggest to better manage cancer in a person with an intellectual disability?
Interview Guide – Family
1. How did the cancer diagnosis unfold?
2. How was the treatment plan initiated?
a. Did the treatment proceed as planned?
b. In your opinion, what issues may have complicated the treatment process?
c. Do you feel the timelines were different compared to in the general population?
3. How were the interactions with the medical teams?
a. How did the medical team adapt to the patient’s disability?
b. How were the patient’s requests and complaints received, understood, and addressed?
c. Were the patient’s routines, fears, phobias, and anxiety-provoking situations considered?
4. Do you feel that the patient’s care pathway was altered due to their disability (psychological limitation)?
a. Were any specific measures implemented to facilitate the patient’s care?
b. What type of support did you receive?
5. What adjustments do you think are important to address the difficulties you encountered?

Note: Five main questions were asked for each guide. Sub-questions were used to help participants develop their thoughts and share their experiences

### Ethics

This study was conducted in accordance with the principles outlined in the Declaration of Helsinki for all human or animal experimental investigations. Informed consent was obtained from all included participants. The study was conducted in accordance with the French Data Protection Commission (CNIL), and all procedures were performed in accordance with the relevant guidelines and regulations. No ethical approval by an independent ethics committee was required at the time of the study conduction, as confirmed in 2024 by the President of the Committee for Personal Protection Sud-Med 4. Hence, at the time of the study’s design and data collection, the authors were required to provide a clear description of the data collected and to store it securely, but no formal approval from an independent ethics committee was required regarding the study’s objectives and procedures.

Given the lack of formal committee approval, particular attention was paid to ethical and conceptual reflexivity. The recruitment strategy was designed to respect participants’ autonomy and minimize potential harm, and all interview data were anonymized to ensure confidentiality. An information letter was provided to participants, and oral consent was obtained, as required by ethics committees for studies including patients. In addition, the researchers explicitly considered power dynamics between researchers and participants. For instance, professionals and family members may hold differing perspectives and degrees of authority in the care of individuals with ID, which could influence what they felt comfortable sharing. The interviewers adopted a reflective approach to minimize the influence of their own positionality, ensuring that participants’ voices were accurately represented. Procedures were carefully structured to mitigate potential coercion, encourage open discussion, and acknowledge the vulnerability of participants, particularly in relation to sensitive topics concerning care practices, patient experiences, and institutional settings.

### Data analysis

Data were analyzed by four researchers (AC, DS, HC, and SHH) using thematic analysis, as described by Braun and Clarke [40]. A mixed qualitative approach was adopted, which combined deductive and inductive strategies.

Deductive coding was guided by predefined codes based on the research objectives, clinical experience, and existing scientific literature, thereby ensuring alignment with the study’s aims. Table 2 presents the codes predefined by our team for use during the analysis. This approach allowed the research team to systematically explore known areas of interest, while providing a structured starting point for analysis. In parallel, inductive coding was applied to allow the emergence of new codes and themes directly from the data, which provided the flexibility to capture unanticipated insights, and to preserve the richness of participants’ experiences. The researchers independently coded a subset of the data to establish initial codes, which were then collaboratively discussed and refined to ensure consistency and reliability. Themes were identified through iterative rounds of analysis, with repeated comparisons of data segments across participants, and triangulation among the four researchers (AC, DS, HC, and SHH) to minimize bias and validate interpretations. This process balanced deductive and inductive approaches, and thus ensured that the final themes and sub-themes were derived from the data itself, rather than being constrained by the predefined codes. This comprehensive coding strategy facilitated the attainment of a nuanced and in-depth understanding of the challenges and experiences reported by participants, while maintaining transparency, consistency, and methodological rigor (see Table 2).

**Table 2:** List of codes related to barriers to care, generated before the analysis

A. Obstacles related to patients
Inability to undergo treatment
Modified treatment/Change in treatment
Non-agreement to treatment
Physical difficulties
Associated illnesses
Psychiatric difficulties/Associated psychiatric conditions
Lack of understanding of the illness
Fear of the unknown
Lack of trust
Struggles with constraints
B. Obstacles related to healthcare providers
Communication difficulties
Lack of knowledge about the patient
Lack of knowledge about the patient’s cancer
Lack of knowledge about intellectual disability
Absence of a point of contact at the hospital
Fear of the patient’s reactions
Monitoring of side effects
Treatment adherence issues
Absence of therapeutic guidelines
Late-stage cancer diagnosis
Insufficient time to dedicate to the patient
C. Obstacles related to professional caregivers
Absence of a caregiver
Lack of knowledge about cancer
No communication with oncology teams
Lack of institutional human resources
D. Obstacles related to family caregivers
Absence of a caregiver
Initial contact issues
Preparation of the care pathway
Support throughout the care journey
Poor communication between professional caregivers and oncology teams
Socio-economic constraints
Ineffective or problematic caregiving

The recruitment process ended when data saturation was reached (i.e., no new information or codes or themes emerged from the participants discourse) [41]. Saturation was assessed iteratively during data collection and analysis: after each set of interviews, transcripts were coded, and emerging themes were compared with those identified in previous interviews. Recruitment continued until no new codes, themes, or substantive insights were observed in successive interviews, indicating that additional data were largely repetitive or confirmatory rather than contributing novel information. This process ensured that the data collected provided a comprehensive understanding of participants’ perspectives while maintaining methodological rigor.

It should be noted, however, that at the time of manuscript preparation, the authors more closely align with the concept of information redundancy [42], which emphasizes the repetition of ideas and confirmation of existing themes rather than the identification of entirely new codes. From this perspective, additional interviews may still provide further details, but these are essentially repetitive or confirmatory, reinforcing previously identified patterns rather than generating novel insights. This may lead to a difference regarding the number of interviews required to achieve data saturation versus information redundancy.

Several strategies were implemented to ensure the rigor and trustworthiness of the study. Credibility was strengthened using researcher reflexivity—including bracketing practices (e.g., use of reflective journals and regular debriefing sessions)—which enabled the team to critically examine and set aside personal biases and assumptions during data analysis. Additionally, peer debriefing and iterative discussions ensured that the interpretations remained grounded in the participants’ accounts. To enhance transferability, a detailed description of the research context, participants, and study setting was provided, thereby allowing readers to evaluate the applicability of the findings to other contexts. Dependability was addressed by maintaining a transparent and detailed audit trail of the research process—including methodological decisions, data management procedures, and coding strategies—which enables replication and external evaluation of the analytic process. Finally, to ensure confirmability, reflexive notes were documented and an audit trail linking the data to interpretations was maintained, thereby reducing the influence of researcher bias, and supporting the neutrality of the findings.

Notably, this study was primarily conducted by individuals who were performing qualitative research for the first time (SHH, HC, and DS; whereas AC had expertise in qualitative research), under the supervision of two experts on qualitative research in health psychology (FCG and KL). Briefly, the authors possess expertise in health psychology (AC, FCG, and KL), psycho-oncology (FCG and KL), and the care of people with ID (SHH, HC, and DS).

## Results

### Participants’ characteristics

**Fig. 1:**
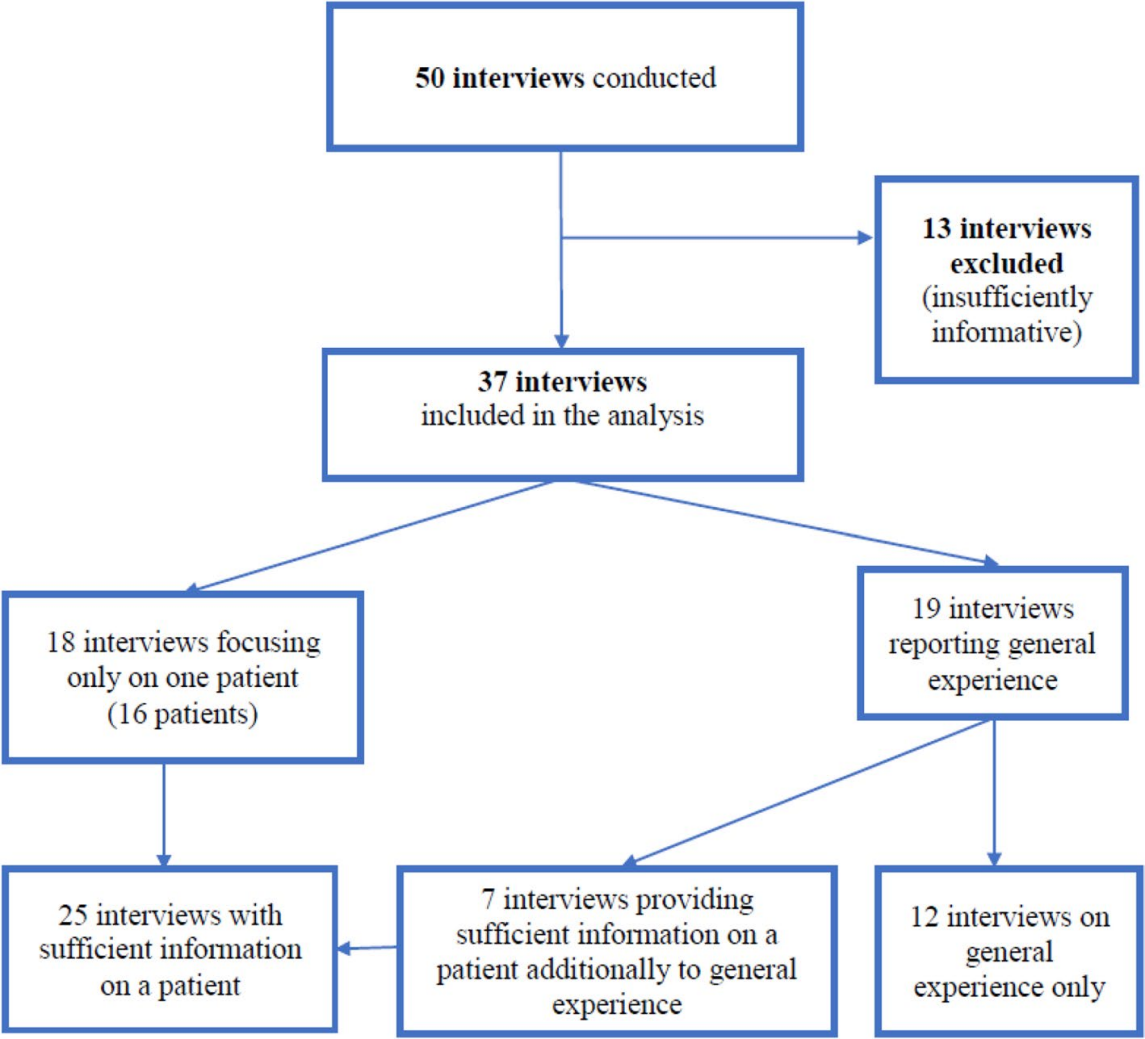
Flow chart of participants inclusion

A total of 50 interviews were conducted to achieve data saturation. Recruitment continued within each participant group (*n* = 28HCPs, *n* = 12 family members, *n* = 10 professional caregivers) until no new themes or substantive information emerged, indicating that data saturation had been reached. This relatively large number reflects both the diversity of participants and professions included, as well as the number of interviews that were subsequently excluded. Thirteen interviews were excluded from the analysis, because insufficient information was provided about the patient with cancer and ID. Some participants were overly reserved or had been inappropriately recruited (i.e., they met the inclusion criteria but had limited experience in caring for people with intellectual disabilities), such that their interviews provided minimal relevant information for analysis. Hence, the final sample included 37 interviews (Fig. 1).

The participants included 17 HCPs (10 HCPs working on oncology teams, and 7 HCPs working in retirement homes or other), 11 family members (5 mothers, 2 fathers, 2 sisters, and 2 brothers), and 9 professional caregivers. Specifically, the included professionals were 10 physicians, 8 nurses, 4 caregivers, 2 directors, 1 coordinating physician, and 1 palliative care nurse. The interviews lasted a mean of 37 min (range: 9 min and 1 h 57 min) and were conducted in-person (*n* = 31, 84%) or by phone (*n* = 6, 16%). A total of 620 pages of transcribed interview data were analyzed.

In 12 interviews, general experiences with cancer treatment among people with ID were discussed. In the other 25 analyzed interviews, information was only provided about a single patient (*n* = 18) or sufficient information was provided about one patient among several patients (*n* = 7). These 25 interviews related to 23 specific patients with ID and cancer (two interviews covered the same individual)—including nine women and 14 men, with ages of 21 to 60 years. Among these patients, diagnoses included mild ID (*n* = 3), moderate ID (*n* = 2), severe ID (*n* = 2), ID without other specification (*n* = 9), Down syndrome (*n* = 3), fragile X syndrome (*n* = 1), autism (*n* = 1), and other genetic conditions (*n* = 2). The most frequently reported malignancies were breast cancer, lymphoma-leukemia, testicular cancer, and malignant melanoma (Table 3).

**Table 3:** Cancer types discussed in 23 interviews that provided precise cancer diagnoses

**Cancer type**	**Family members**	**Oncology teams**	**Professional caregivers**	**Retirement home workers and others**	**Total**
Breast cancer	2		4	2	8
Lymphoma, leukemia	3	1			4
Testicular cancer	2		1		3
Melanoma	1	1	1		3
Colorectal cancer				2	2
CNS tumor	1				1
Pancreatic cancer	1				1
Endometrial cancer				1	1
Total	10	2	6	4	**23**

*CNS* Central nervous system

### Themes identified

Theme 1 includes every barrier and difficulty in obtaining adequate care that was described by the participants. Theme 2 includes the events and circumstances that facilitated successful cancer treatment.

### Theme 1: barriers to cancer care for people with cancer and intellectual disability

Participants reported several barriers, which were all interconnected. The main barriers were related to patients’ limitations, communication difficulties, professionals’ lack of knowledge, and delayed cancer diagnosis. Table 4 presents the various barriers identified from the participants’ discourses.

**Table 4:** Barriers to cancer care for people with cancer and intellectual disability (ID)

Theme 1. Barriers to cancer care	Representative quote [free translation from verbatim French]
Patient’s limitations	
Psychological limitations	“Either we tend to eliminate the deficient patient by saying ‘no, he cannot benefit from treatment’, perhaps wrongly, or on the contrary, we impose treatment on him, which can be an aggression or experienced as an aggression” *(PART 45, Hematologist)*
Biological limitations	“…We decided not to do chemotherapy, because chemotherapy is not compatible with a person like that.” *(PART 43, Medical oncologist)*
Treatment non-agreement	“If there really is a categorical non-agreement, and we feel that it really is … that poses the problems of stress, anxiety, and refusal. Non-agreement is associated with other things. It’s going to be ‘I put myself in a corner; I don’t want to eat’ … So that’s really much more important.” *(PART 6, Nurse)*
Lack of knowledge	
About ID among oncology teams	“So it obviously goes, you have to know that certain manifestations are in fact the expression of pain. I think we are not always trained enough for that.” *(PART 43, Medical oncologist)*
Physician refusal of treatment	“There is significant reluctance because they see the injection, or because when we do a scan, he doesn’t want to go under the arch […]” *(PART 33, Urologist)*
About cancer among caregivers	“We too have little understanding of cancer.” *(PART 35, Institution director)*
Communication difficulties	
Physician – Patient	“… we don’t have any communication that allows us to say exactly what she’s thinking, and if she realizes the seriousness of things.” *(PART 5, Medical oncologist)*
Oncology teams – Retirement home workers	“Afterwards, if we don’t ask for news, they don’t give us any so … But then we haven’t been told anything …” *(PART 11, Nurse working in a retirement home)*
Oncology team – Institutions	“Effectively, being really in touch with the healthcare services, so that they can give us good advice, because we here are not in healthcare, so there are things we don’t know.” *(PART 28, Educator)*
Late cancer diagnosis	“… and as a result, this will delay the start of screening, and this may also affect the treatment itself. It is common for symptoms of illness to be detected later.” *(PART 32, Educator and team leader)*
Absence of professional caregiver	“The caregiver in consultation is really essential, because otherwise the dialogue is not stimulated” *(PART 40, Medical oncologist)*
Insufficient time to ensure patient understanding	“We, people we see for 20 min, half an hour from time to time, it’s complicated to know a little bit about how they plan for the future.” *(PART 46, Dermatologist)*
Lack of staff or family caregiver	“In outside consultations, there is no one; we do not accompany residents outside.” *(PART 9, Nurse working in a retirement home)*
Difficult attitude of cancer professionals	“She shouldn’t move for a whole day. She was scolded for moving a little, and she got tired of it after a while.” *(PART 20, Mother)*
Difficulties with professional caregivers	“… I think they could have been a little better with the information … I wasn’t aware, what happened?” *(PART 37, Father)*
High cost of care	“…It was a private clinic; it had a cost, a phenomenal cost. In addition to what was reimbursed by social security, this story of cancer over a year costed [cost amount]” *(PART 48, Sister)*

Most of the reported barriers to care were limitations related to the patient—including psychological limitations (e.g., fear of white coats, and fear of “big machines”), difficulty evaluating symptoms (e.g., pain, and adverse effects of the treatment), and biological characteristics that limited the use of chemotherapy or radiotherapy. For example, one oncologist explained “We decided not to do chemotherapy, because chemotherapy is not compatible with a person like that.” (PART 43). Additionally, treatment non-agreement from the patient was perceived as a barrier to care, associated with the difficulty of having discussions with the patients. Treatment non-agreement was often due to fear, mistrust, or lack of understanding, representing another layer of complexity of care. One HCP recalled that a patient with schizophrenia refused lung cancer surgery, stating that his body was perfect. He insisted that it was out of the question to remove any part of it. Another participant reported that a patient with schizophrenia refused further treatment after the removal of one breast; although she had agreed to the operation, she may have not fully understood what was being done.

The second most commonly reported barrier encompassed communication challenges across various levels. Difficulties in communication between oncologists and patients were highlighted, particularly in terms of adapting medical explanations to the cognitive level of people with ID. This often led to misunderstandings, the provision of incomplete information, or treatment non-agreement, as explained above. Additionally, communication gaps between the oncology team and professional caregivers were cited as a significant barrier, as illustrated in the following quote from a nurse: “Afterwards, if we don’t ask for news, they don’t give us any so…” (PART 11). These gaps often resulted in inconsistent care plans, or delays in decision-making. Finally, participants noted barriers when collaborating with professionals in retirement homes and other structures, where staff may lack oncology-specific knowledge, or experience in managing patients with complex needs. Such multilayered communication barriers hindered the continuity of care, and created additional stress for HCPs, who struggled to ensure alignment across all stakeholders.

Participants also reported a barrier to care related to oncology team members’ lack of knowledge about ID, which often led to insufficiently adapted care strategies or communication. Medical oncologists indicated that they were not prepared to treat people with ID. Two HCPs expressed regret over the lack of guidelines and adapted protocols for people with ID, in terms of chemotherapy and radiotherapy. On the other hand, participants highlighted a lack of cancer-specific knowledge among professional caregivers working in ID institutions, which led to difficulties in recognizing symptoms, managing treatments, and providing adequate support during the care process. Accordingly, an institution director stated: “We too have little understanding of cancer.” (PART 35). The participants reported that these knowledge gaps on both sides contributed to fragmented care, and heightened challenges in coordinating effective treatment plans. Additionally, nurses and oncology nurses indicated the need for training focused on working with people with ID, to prepare them for complex and challenging situations.

Another barrier reported by the participants was the discovery of cancer already at an advanced stage. Participants noted that late diagnosis often stemmed from delayed recognition of symptoms, due to either communication difficulties with patients who do not express their symptoms (e.g., who may feel pain but not talk about it), or a lack of routine screening adapted for individuals with ID. Delayed diagnoses frequently limited the available treatment options and reduced the likelihood of successful outcomes.

Other obstacles were less frequently mentioned by participants, including those related to systemic and logistical challenges in the provision of cancer care for people with ID. Some participants reported a lack of time among oncology teams, often leading to rushed consultations making it difficult to provide individualized explanations or to ensure patient understanding. Similarly, professional caregivers commonly struggled to find time to accompany patients to treatments, further hindering care continuity. One nurse noted the following: “In outside consultations, there is no one; we do not accompany residents outside.” (PART 9). Another described barrier was the shortage of adequately trained staff in retirement homes, which made it difficult to provide consistent support for patients. Additionally, some family members highlighted inappropriate attitudes among some HCPs in oncology—for example, a lack of consideration, lack of patience to listen to the patient, not considering the patient’s input, or treating the patient as if they had no ID—which occasionally undermined trust and patient cooperation. Other reported barriers included treatment non-agreement by some physicians, often due to concerns that the risks may outweigh the benefits in this vulnerable population, and the high cost of care (i.e., related to care in private clinics or to see specialists outside of the hospital), which placed substantial financial strain on some family members.

### Theme 2: facilitators of cancer care for people with cancer and intellectual disability

Analysis of the participants’ accounts using an inductive approach identified four key factors that positively influenced cancer care for people with ID: a proactive attitude from caregivers, consistent presence of caregivers throughout the cancer journey, the patient having a positive attitude, and a tailored environment (see Table 5).

**Table 5:** Facilitators of cancer care for people with cancer and intellectual disability

Theme 2. Facilitators to cancer care	Representative quote [free translation from verbatim French]
Proactive attitude from family members or professional caregivers	“Well, someone who knows the person will know how they reacted and what it is, their situation, their different way of presenting themselves, and how we can help. More than us, who will judge on a different basis.” *(PART 33, Oncologist)* “So, actually, having a caregiver is really helpful—someone they know and trust—it really reassures them. They need this.” *(PART 34, Oncologist)*
Presence of family members or professional caregivers	“It was a very deep and short anesthesia, but I was next to my brother until he was anesthetized. I was in the next room; I was notified as soon as he opened one eye in the recovery room. I was able to be near him for this short anesthesia.” *(PART 48, Sister)* “That is to say that they often come with a nurse coordinator, which is great. We often have the patient’s referring nurse with us, who comes to the consultation and accompanies them with their legal representative. This is what we are trying to put in place. It’s a lot of people but it’s true that there are really close people who can summarize it well.” *(PART 34, Oncologist)*
Patient’s positive attitude	“Well, it will be more the positive side, because the innocence of his handicap meant that he was not aware of the seriousness. Obviously, the less we worry, the more we can overcome and continue.” *(PART 23, Mother)* “For people to trust you again, we spend days there, we spend months there … even this person, despite all their aggressiveness at the start and everything … they trusted us.” *(PART 11, Nurse working in a retirement home)*
A tailored environment	
Tailored reception at the hospital	“And she immediately felt at ease. And, so, she was well-received. In fact, for O., the most important thing is that she feels well-surrounded by the people she is used to seeing.” *(PART 37, Mother)*
Tailored organization at the hospital	“That is to say, we have dedicated consultation slots, which are quickly available. I have every Tuesday morning where the consultations are not resolved; they serve as announcement consultations. I always have an hour or two in my schedule for that.” *(PART 34, Urologist)*
Preparation and anticipation	“So, we do empty tours; that is to say, we put them in the room, we don’t do radiotherapy, we show them. But the radiologists must have the time, we need available places. We do this for the children, we do it for them as well.” *(PART 33, Oncologist)*

A proactive attitude from family members or professional caregivers, particularly mothers, was deemed essential. An oncologist stated the following: “So, actually, having a caregiver is really helpful—someone they know and trust—it really reassures them. They need this.” (PART 34). Such caregivers are perceived as playing a pivotal role in navigating the healthcare system. They can provide the care team with valuable information about the patient, help navigate through the healthcare system, facilitate smoother transitions between appointments, and ensure timely interventions. Additionally, participants perceived the caregivers as providing emotional and practical support to patients, helping the patients feel reassured and better prepared to face challenges. Participants also indicated that the caregivers’ knowledge of the patients was a primary factor that facilitated care management.

The presence of a family member or professional caregiver throughout the cancer journey was also highlighted as a crucial factor. For example, a sister described being present during anesthesia, stating “I was able to be near him for this short anesthesia.” (PART 48). This presence was perceived as being helpful to provide stability, foster trust, and act as a bridge between the patient and HCPs. It was specifically identified that this presence could help ensure that the patient’s needs and preferences were consistently communicated and addressed. Moreover, our interviews suggested that family members or close friends who had also undergone cancer treatment could have a positive impact on patient compliance. For example, a mother who was previously treated for breast cancer was able to help her daughter with ID overcome fears of radiotherapy machines.

Participants also reported that the patient having a positive attitude themselves significantly contributed to the effectiveness of care. It was noted that patients who exhibited confidence and maintained a strong trusting relationship with their familial or professional caregivers, were more cooperative and resilient during treatment. Notably, trust is built on a long relationship, as one nurse stated: “For people to trust you again, we spend days there, we spend months there” (PART 11). It was perceived that a patient having a positive and trusting attitude enhanced the patient’s overall experience and engagement with care.

Finally, participants emphasized that a tailored and welcoming environment significantly facilitates the care of people with ID. They highlighted several key factors, including the importance of a tailored reception at the hospital, ensuring that initial interactions are inclusive and supportive. Participants also mentioned the need to adapt communication, emphasizing that patients should be reassured through clear and compassionate dialogue. Respecting individual needs and adapting care approaches were described as fundamental principles. For this reason, consultations with people with ID were frequently longer than usual, with one urologist stating: “I always have an hour or two in my schedule for that.” (PART 34). Furthermore, the organization of the hospital played a pivotal role, with participants emphasizing the value of preparation and anticipation for effectively managing appointments, procedures, and patient-specific requirements. For example, some participants reported streamlining the care pathway by reserving consultations slots, eliminating waiting times for specific procedures, and implementing customized arrangements. These elements were identified as instrumental for creating an environment conducive to high-quality equitable care.

## Discussion

The present study highlights the key facilitators who support the cancer care journey of people with ID, providing novel insights that complement the existing literature regarding barriers to care. While the elucidated obstacles—such as communication difficulties, limited knowledge about ID, and diagnostic delays—highlight ongoing challenges in providing equitable care, our findings particularly underscore positive factors that can improve care. These include proactive and engaged attitudes from family members and professional caregivers, the consistent involvement of caregivers throughout the care pathway, the patient having a positive outlook, and tailored care environments. By focusing on these facilitating factors, alongside recognized barriers, our study offers practical guidance for the development of interventions aimed at improving cancer care for individuals with ID.

Importantly, although the interviews were conducted between 2017–2018, the relevance of these findings persists today [43, 44]. Structural and systemic issues identified in the study—such as the persistent lack of formal training in ID among oncology professionals, the continued absence of widely implemented adapted oncology guidelines, and ongoing risks of diagnostic delay—remain largely unchanged in France and in several countries. While developments such as increased awareness of disability rights and more general attention to inclusive care practices have occurred, these have not fundamentally altered the structural constraints and organizational challenges highlighted by participants. This reinforces the continued applicability of our findings for informing interventions and policy aimed at improving cancer care for people with ID.

A main contribution of this study lies in identifying factors that facilitate care. Only scarce literature mentions facilitating factors for the provision of cancer care to people with ID. We could not identify any study focused on this question. However, we found some information drawn from different approaches. Tuffrey-Wijne et al. [18] summarized six points that help adults with ID in their cancer journey: 1) the presences of trusted family or a paid carer,2) preparation for appointments; 3) explanation of procedures in a way that is easy to understand; 4) familiarity with the hospital or clinic; 5) routines, old or new; and 6) a hospital staff sensitive to the patient’s needs. All of these points were identified in our participants accounts, except that routines were not reported. Additionally, two previous studies highlighted the need to prepare physicians [45] and nurses [36] to develop skills for meeting the needs of persons with ID. Indeed, a supportive environment [14], with professionals trained to accommodate ID, is essential. However, it is not always feasible to implement such a supportive environment in oncology settings, due to resource constraints and limited training. Nevertheless, highlighting and promoting a positive environment—even when full specialist support is unavailable—can help guide practical strategies for healthcare teams to enhance patient-centered care and reduce disparities.

The international literature contains more information regarding barriers to care. Most studies of this topic have been conducted in Australia and the UK (e.g., [28, 39, 45–47]) and have reported findings consistent with ours. This indicates that, regardless of the care context or country, people with ID face similar challenges in accessing and receiving cancer care. The main obstacles identified in the present study relate to cognitive, psychological, and physical limitations, which may lead patients to refuse exams or treatments (including imaging or chemotherapy), and can complicate symptom evaluation, particularly in terms of pain, fatigue, or appetite loss [26, 29, 48]. Care is further hindered by communication difficulties, since effective interaction between patients, caregivers, and oncology teams is crucial for decision-making, symptom assessment, and treatment success [25, 39, 45, 46, 49, 50]. Establishing therapeutic relationships with patients—supported by caregivers who can interpret subtle behavioral cues—requires respectful, slow, and simple communication, along with verification of understanding [39, 50]. Another barrier identified in our study is the lack of knowledge about ID among HCP, which affects symptom evaluation, communication, and treatment decisions. This has previously been reported in the literature [45, 49]. Another substantial barrier mentioned in the literature is delayed diagnosis, resulting in larger and more advanced tumors [21, 47, 51–53]. Diagnosis can be delayed because people with ID often cannot effectively communicate symptoms, and behavioral changes may be misinterpreted as ID-related rather than indicative of illness [37, 54]. It was somewhat surprising that some previously reported barriers were not identified in our participants accounts—for example, inability to undergo treatment, the patient’s lack of knowledge, and treatment adherence [17, 25, 39]. This does not imply that such barriers do not exist, but rather suggests that they may not have been explicitly perceived or prioritized by the participants in this study.

### Recommendations

The present findings provide valuable insights to inform educational programs, clinical guidelines, and care coordination for people with ID who are undergoing cancer care:

- Targeted training programs for HCP—including general practitioners, oncologists, nurses, and residential caregivers—should focus on improving knowledge of ID, effective communication strategies, symptom assessment, and individualized care planning, as previously formulated [45, 49, 55].
- Clinical guidelines should incorporate specific recommendations for adapting diagnostic procedures, treatment protocols, and follow-up care to accommodate cognitive, psychological, and physical limitations, ensuring safe and effective care for this population. Efforts should be made to implement supportive environments—such as tailored communication tools, quiet and familiar settings, and flexible scheduling—to reduce patient anxiety [56] and enhance engagement in care.
- Multidisciplinary team organization: Healthcare teams should solicit professionals with potential expertise in ID care—namely psychologists, social workers, and nurses—to enable collaborative decision-making, comprehensive support, and more effective care planning across the cancer pathway. When no team member has the necessary expertise, the team should consult professionals outside oncology who specialize in supporting people with ID, seek assistance from relevant organizations, or include family members to better understand patients’ needs and preferences.
- Care coordination should actively involve family members and professional caregivers, recognizing their critical role in interpreting patients’ behaviors, supporting communication, and facilitating adherence to treatment.

Additionally, policymakers and healthcare institutions should prioritize systematic monitoring of cancer care delivery for people with ID, promoting early diagnosis and timely interventions to reduce disparities and improve outcomes. Collectively, these recommendations could enhance equity, quality, and patient-centeredness in the provision of cancer care for people with ID.

### Strengths and limitations

One strength of the study is that we interviewed different actors in cancer treatment (i.e., data triangulation)—including not only members of oncology teams, but also professionals and family caregivers who are in close contact with the patients and have a better understanding of how treatments impact the patient. We included both physicians and nurses from oncology teams, to collect different points of view. Similarly, families offer different and complementary approaches; and physicians, nurses, caregivers, psychologists, and directors from institutions complete the pool of experiences. Another key strength of this study is the use of investigator triangulation, with multiple members of the research team independently analyzing the data and collaboratively discussing themes. This practice minimizes researcher bias and improves the reliability of the results by obtaining consistent findings and reaching consensus [57].

A limitation of this study is that the sample is likely not representative of all people with ID treated for cancer, since persons living with their families or alone were underrepresented. Patients were exclusively recruited through the Oncodéfi association, which may limit the generalizability of the findings to individuals with ID receiving cancer care outside of this network. Nonetheless, the number of analyzed interviews, the diversity of cancers represented in the sample, the range of patient ages, and the various places where patients lived amounted to a wide spectrum of therapeutic situations. Another limitation is that some people with ID may not have received any treatment—for example, if their disease stage was considered beyond therapeutic possibilities. Additionally, patients were not directly interviewed, due to challenges related to the patients’ understanding of their treatment [37]. Moreover, the analyzed data are somewhat dated,however, this is unlikely to have a significant impact, since the findings appear to reflect the current realities of clinical practice in this field. Another possible limitation is that the interviewer’s preunderstanding and the research team’s prior experience may have influenced the analysis and interpretation of the results. Finally, the interview guide developed and used in the study may have directly influenced the data collected and the results obtained, as it framed the topics discussed and the depth of information shared by participants. Conducting open interviews could have been useful to allow participants to freely explore their experiences beyond the topics framed by the interview guide. Overall, our findings represent a first step in understanding the landscape of cancer treatment among people with ID. Further studies are needed to confirm these results, explore additional perspectives, and identify strategies to continue to improve care and support for this population.

## Conclusion

The present study provides insights into cancer treatment among a large sample of adults with ID. Our findings represent the experiences of oncology teams, families, and professional caregivers in assisting people with ID in cancer treatment. Our participants’ accounts confirmed barriers to care, and suggested ways to improve cancer treatment for people with ID. This work highlights the value of further evaluating the presently identified barriers to care, such as the need for physician and nurse education about ID, the need for improved communication between oncology teams and caregivers without knowledge of cancer, and delayed cancer diagnosis in people with ID. Importantly, our findings suggest that many obstacles and difficulties can be alleviated by improving these factors.

## Supplementary Information

Supplementary Material 1.

## Data Availability

The datasets collected and analyzed during the current study are not publicly available due to French regulations (RGPD), but are available from the corresponding author on reasonable request.

## Abbreviations

HCP: Health Care Professionals
ID: Intellectual Disability
