# Supplementary material for: Navigating cancer treatment in people with intellectual disability: a qualitative study of professionals’ and family members’ perspectives
Source: BMC Cancer. 2026 Jan 30;26:310. doi: 10.1186/s12885-026-15601-6 (PMC12947367; doi:10.1186/s12885-026-15601-6)
Supplement: Supplementary file 1 — Supplementary Material 1. [file 12885_2026_15601_MOESM1_ESM.docx]

**Appendix 1.** Ethical approval exception.

Montpellier, le 25 mars 2024

**QUALIFICATION DE LA RECHERCHE**

**« Obstacles aux soins chez les personnes déficientes intellectuelles atteintes de cancer »**

Cette recherche a été réalisée entre 2017 et 2018 en collaboration avec l’association Oncodéfi et Épidaure - Département de Prévention de l’institut régional du Cancer de Montpellier. C’est une étude qualitative qui comprend des entretiens semi-directifs. Elle a pour finalité d’évaluer la prise en charge de patients déficients intellectuels atteints par des professionnels de santé et des membres de la famille. Elle inclut 25 soignants ayant tous pris en charge, au moins une fois au cours de leur carrière, des patients déficients intellectuels atteints d’un cancer et 12 proches de personnes déficientes intellectuelles ayant eu un diagnostic de cancer.

Selon l’article R1121-1 du code de la santé publique point II, 2° « Ne sont pas des recherches impliquant la personne humaine au sens du présent titre les recherches qui ne sont pas organisées ni pratiquées sur des personnes saines ou malades et n'ont pas pour finalités celles mentionnées au I, et qui visent à évaluer des modalités d'exercice des professionnels de santé ou des pratiques d'enseignement dans le domaine de la santé. »

Cette recherche n’est donc pas une recherche impliquant la personne humaine tel que défini par la règlementation française. Au moment de la réalisation de l’étude, celle-ci ne relevait donc pas de l’avis d’un comité éthique indépendant.

Virginie Rage

Présidente Comité de Protection des Personnes (CPP) Sudmed4

[Authors free English translation]

Montpellier, March 25, 2024

**QUALIFICATION OF THE RESEARCH**

**“Barriers to care for individuals with intellectual disabilities and cancer”**

This research was conducted between 2017 and 2018 in collaboration with the Oncodéfi association and Épidaure - Prevention Department of the Regional Cancer Institute of Montpellier. It is a qualitative study comprising semi-structured interviews. The main aim of the study was to evaluate the care provided to patients with intellectually disability by healthcare professionals and family members. The study includes 25 healthcare professionals, all of whom had, at least once in their careers, cared for patients with intellectual disability and cancer, and 12 family members of individuals with intellectual disability and cancer.

According to Article R1121-1 of the French Public Health Code, paragraph II, section 2: "Research that is not organized or conducted on healthy or ill individuals and does not aim for the objectives mentioned in paragraph I, but rather seeks to evaluate healthcare professionals’ practices or teaching methods in the field of healthcare, does not qualify as research involving human subjects as defined in this title."

Thus, this research does not fall under the category of research involving human subjects as defined by French laws. At the time of the study's execution, it was therefore not subject to review by an independent ethics committee.

Virginie Rage

President of the Ethics Committee for the Protection of Persons (CPP) Sudmed4
